# Effects of Dietary *Alpinia officinarum* Extract on Performance, Immunity, Microbiota, and *Aeromonas hydrophila* Resistance in Red Claw Crayfish

**DOI:** 10.3390/ani16162525

**Published:** 2026-08-13

**Authors:** Zi-Hang Yu, Yao-Peng Lu, Chi Xu, Xiu-Xia Zhang, Ze-Long Zhang, Pei-Hua Zheng, Jun-Tao Li, Jun-Feng Tian, Hui Guo, Jian-An Xian

**Affiliations:** 1 Guangdong South China Sea Key Laboratory of Aquaculture for Aquatic Economic Animals, College of Fisheries, Guangdong Ocean University, Zhanjiang 524025, China; 2 Hainan Provincial Key Laboratory for Functional Components Research and Utilization of Marine Bio-Resources, Institute of Tropical Biosciences and Biotechnology, Chinese Academy of Tropical Agricultural Sciences, Haikou 571101, China

**Keywords:** plant extract, crustaceans, non-specific immunity, gene expression, disease resistance

## Abstract

Plant extracts contain multiple bioactive compounds and have been extensively investigated as alternatives to antibiotic-based feed additives in aquaculture. The present study was designed to evaluate the effects of dietary supplementation with galangal (*Alpinia officinarum*) extract (AO) on the growth, immune response, intestinal digestive enzyme activities, gut microbiota, and resistance to *A. hydrophila* in red claw crayfish (*Cherax quadricarinatus*). Crayfish with an initial body weight of 0.83 ± 0.05 g were randomly assigned to 18 tanks (6 groups × 3 replicates) and were fed basal diets supplemented with 0, 0.5, 1, 3, 5, or 7 g/kg of AO for 8 weeks. The weight gain rate (WGR) and specific growth rate (SGR) of crayfish were significantly higher in the 3 g/kg and 5 g/kg groups than in the control group. Dietary supplementation with 3–5 g/kg AO significantly elevated the crude protein content and reduced the crude lipid content in the muscle. AO supplementation at 3–5 g/kg significantly enhanced the total antioxidant capacity (T-AOC) and the activities of glutathione peroxidase (GPx), catalase (CAT), acid phosphatase (ACP), and phenol oxidase (PO), whereas the content of malondialdehyde (MDA) was significantly reduced. Dietary AO supplementation markedly increased the relative abundance of Firmicutes, *Cytophaga*, and *norank_o_Saccharimonadales*. Moreover, dietary AO significantly upregulated the expression of multiple functional genes in the hepatopancreas, including *ecCu, Zn-SOD*, *Trx1*, *ALF*, *C-LZM*, *GPx*, and *Se-GPx*, and improved the survival of the *C. quadricarinatus* under challenge with *A. hydrophila*. Collectively, the findings of the present study indicate that dietary supplementation with 3–5 g/kg AO enhances the growth performance, intestinal digestive enzyme activities, immune function, and disease resistance in the red claw crayfish (*C. quadricarinatus*).

## Simple Summary

Galangal (*Alpinia officinarum* Hance) is one of the most economically important species of the genus, valued for both its medicinal and culinary applications, and is widely cultivated in several provinces in China. This study aims to evaluate the effects of dietary supplementation with *Alpinia officinarum* extract (AO) on growth, immune response, intestinal digestive enzyme activities, gut microbiota, and resistance to *Aeromonas hydrophila* in red claw crayfish (*Cherax quadricarinatus*). Dietary supplementation with AO enhanced the growth performance, immunity, and disease resistance of crayfish. Dietary AO also was associated with altered composition or enzyme activity in the intestine. Furthermore, AO supplementation induced the expression of immune-related genes in the hepatopancreas. Collectively, these findings highlight that AO may warrant evaluation as a potential phytogenic additive.

## 1. Introduction

Red claw crayfish (*Cherax quadricarinatus*), also known as the Australian freshwater crayfish, is classified within the phylum Arthropoda, subphylum Crustacea, class Malacostraca, and order Decapoda [1]. This species is natively distributed across the freshwater basins of northern Australia and Papua New Guinea, and it ranks among the largest freshwater decapods. Owing to its rapid growth rate and robust environmental adaptability, the red claw crayfish has progressively emerged as an economically important freshwater species for aquaculture [2]. Furthermore, the species is valued for its palatable meat, which is rich in protein and low in lipids, thereby rendering it highly popular among consumers [3]. As the market demand continues to expand, the red claw crayfish exhibits considerable economic value and broad aquaculture prospects [4]. However, one of the major causes of mass mortality in red claw crayfish is the rapid proliferation of opportunistic pathogens, such as *Aeromonas hydrophila*, within aquaculture ecosystems [4]. *A. hydrophila* is widely distributed across freshwater, seawater, and soil. The bacterium can infect aquatic animals through wounds, the respiratory tract, or the digestive system, thereby causing a variety of diseases in farmed animals, including septicemia, gill disease, and enteritis [5,6,7].

As aquaculture continues to expand, the drawbacks of intensive farming have become increasingly apparent—most notably the outbreak of aquatic diseases and the overuse of antibiotics driven by outdated production conditions and technical practices [8,9]. The mainstream flow-through and recirculating aquaculture systems typically require substantial capital investment and operational expenditure [10], and under conditions of inadequate management or high stocking densities, they can experience water quality deterioration that may lead to disease outbreaks in aquatic animals [11]. The routine prophylactic use of antibiotics, combined with heavy chemical disinfection of the farming environments, leads to the accumulation of drug residues in aquatic animals and ultimately poses a risk to human consumers [12,13]. Incomplete disinfection of the aquaculture water, inappropriate antibiotic use that fosters resistance, and residual disinfectants at elevated concentrations collectively compromise the health of aquatic animals [14,15]. Therefore, the development of environmentally sustainable and effective antibiotic-free feed additives has become imperative for the continued advancement of aquaculture.

Plant-based additives are pharmacologically active compounds derived from botanical sources, which are valued for their accessibility, ease of preparation, and cost-effectiveness [16]. These additives are also characterized by low residue levels and minimal toxicity, which result in fewer adverse effects in animal production and contribute to the general safety of food products for human consumption [17]. Furthermore, a substantial body of evidence indicates that plant extracts confer multiple benefits in aquatic animals, including improved growth performance, health, reproductive capacity, and product quality, along with enhanced resilience to environmental fluctuations in aquaculture systems [18,19]. Numerous countries have therefore adopted plant-based additives as effective alternatives to antibiotics, thereby not only reducing the incidence of aquatic animal diseases and lowering the disinfectant usage, but also strengthening the innate immune systems of aquatic animals through diverse mechanisms [20].

Galangal (*Alpinia officinarum* Hance) is a perennial herbaceous species of the genus *Alpinia* in the *Zingiberaceae* family. It is one of the most economically important species of the genus, valued for both its medicinal and culinary applications, and is widely cultivated in several provinces in China, including Guangdong, Hainan, Guangxi, Taiwan, and Yunnan, where it serves as both a culinary spice and a medicinal ingredient [21]. The plant is rich in bioactive constituents; its phenolic compounds, flavonoids, and diarylheptanoid derivatives contribute to a broad spectrum of pharmacological activities, including anticancer, anti-inflammatory, antioxidant, antibacterial, and antiviral effects, along with protection of the cardiovascular and digestive systems [22]. Previous studies have demonstrated that *Alpinia officinarum* extract (AO) can function as an immunostimulant, thereby enhancing the immune responses and modulating gut microbiota [23,24]. Additionally, AO has been reported to enhance the hatchery survival and the survival of juvenile catfish [25] and to improve the gut microbiota composition of *Litopenaeus vannamei* [23]. Furthermore, a study showed that AO encapsulated into mesoporous silica nanoparticles in fish diets as a supplement enhances the immune response and disease resistance of rainbow trout [24]. To date, the effects of AO on crayfish have remained uninvestigated. Therefore, the exploration of its potential roles, particularly in antioxidant activity, immune modulation, and gut microbiota regulation, holds considerable scientific and practical value. The present study evaluated the effects of dietary supplementation with AO on the growth performance, immunity, intestinal digestive enzyme activities, gut microbiota, and disease resistance to *A. hydrophila* in red claw crayfish.

## 2. Materials and Methods

### 2.1. Crayfish Source

The crayfish were procured from a commercial farm in Wenchang, Hainan, China. Prior to the experiment, the crayfish were acclimated in a recirculating aquaculture system for 1 week. The water parameters were maintained at the following ranges throughout the trial: temperature, 25–28 °C; dissolved oxygen, >5 mg/L; pH, 7.5–7.8; total ammonia nitrogen, <0.5 mg/L; nitrite-N (NO_2_^−^-N), <0.1 mg/L; nitrate-N (NO_3_^−^-N), <20 mg/L; and total hardness (as CaCO_3_), 150–200 mg/L. The crayfish used in this trial were too small to allow reliable sex identification prior to the experiment; therefore, sex was not balanced among treatments.

### 2.2. Plant Extracts

The AO used in the present study was prepared at the Institute of Tropical Biotechnology, Chinese Academy of Tropical Agricultural Sciences (Haikou, China). The extraction procedure and component quantification for the AO were performed as described previously [26]. The galangal stems and leaves were crushed and extracted by heat reflux extraction for three times (three extractions of eight, six, and six times the quantity of water for one hour, respectively). The mixture was filtered using Whatman No.1 filter paper. Filtration was carried out to collect the extract and concentrate it in the form of an infusion. The paste extract was mixed with 4 times the amount of anhydrous ethanol (Xilong Scientific, Guangzhou, China) and left to stand for one day. The extract was then strained and concentrated to recover ethanol. The product was oven-dried for 8 h, then dried in a vacuum oven for 6 h, crushed, sterilized and kept at −20 °C for use.

#### 2.2.1. Total Flavonoid and Total Phenolic Content

The AO was evaluated with regard to the total flavonoid content (TFC) by utilizing the aluminum chloride technique [27]. TFC was expressed by rutin equivalent (y = 2.4599x − 0.115, R2 = 0.999). TFC was expressed as mean ± SD (mg rutin equivalent/g dry extract). The extract was evaluated using the Folin–Phenol technique [28]. The total phenolic content (TPC) was expressed by gallic acid equivalent (y = 0.2447x − 0.0013, R2 = 0.999). TPC was expressed as mean ± SD (mg GA equivalent/g dry extract).

#### 2.2.2. LC-MS Analysis

AO samples were taken, lyophilized and mixed with 1000 μL of solution (MeOH: Acetonitrile (ACN): H_2_O, 2:2:1 (*v*/*v*/*v*)). Mixed products were homogenized in a 4 °C water bath (35 Hz, 4 min) and then sonicated for 5 min and repeated three times. The mixture was incubated at −40 °C for 1 h to precipitate proteins, followed by centrifugation at 4 °C (12,000 rpm, 15 min). Liquid (200 uL) was transferred to the wells of a 0.22 μm filter plate. The filter plate was placed on a manifold, and a vacuum was applied (4 psi, 90 s). The plate was removed from the positive-pressure device for analysis. For non-polar metabolites, LC-MS analysis was performed using an ultra-high-performance liquid chromatography (UHPLC) system (Vanquish, Knoxville, TN, USA) equipped with a Phenomenex Kinetex C18 (2.1 mm × 50 mm, 2.6 μm) and an Orbitrap Exploris 120 mass spectrometer (Orbitrap MS, Thermo Fisher Scientific, Waltham, MA, USA). Mobile phase A consisted of 0.01% aqueous acetic acid, and mobile phase B was a 1:1 (*v*/*v*) mixture of isopropyl alcohol (IPA): ACN. The injection volume was 2 μL, and the autosampler temperature was 4 °C. The column temperature was 25 °C. An in-house XCMS-based software was used to locate, extract, align, and integrate peaks from the raw data after it was converted to mzXML format using ProteoWizard (V3.0.26038). The BiotreeDB (V3.0) and R package were utilized to identify the metabolites.

### 2.3. Basic Diet Preparation

The basal diet formulation is shown in Table 1. Six experimental diets were prepared, containing 0 g/kg (CG), 0.5 g/kg (AO1), 1 g/kg (AO2), 3 g/kg (AO3), 5 g/kg (AO4), and 7 g/kg (AO5) of AO, respectively. All the ingredients were thoroughly mixed and processed into a dough. The feed was pelleted to 2.0 mm diameter granules using a small twinscrew extruder (Shanghai Yutian Precision Machinery Production Co., Shanghai, China). All diets were air-dried using a ventilated oven (Shanghai Yiheng Scientific Instrument Co., Shanghai, China) at 40 °C and stored at −20 °C for further use.

### 2.4. Crayfish Management

The feeding trial was conducted in a recirculating aquaculture system. The procedures and protocols for this study have been ethically reviewed and approved according to the guidelines of the relevant institutional committees and granted an Aquaculture Research Permit (ITBB20240601). The system consisted of 18 tanks (500 L), each equipped with an independent water supply and drainage line. Healthy crayfish of uniform size with an initial body weight of 0.83 ± 0.05 g were randomly distributed into experimental tanks (500 L) and assigned to six groups, each with three replicates of 40 crayfish. The aquaculture tanks were arranged in a completely randomized block design. The culture period lasted 8 weeks. Forty PVC pipes (Length: 12 cm) were placed in each tank and glued together to prevent crayfish cannibalism. The crayfish were fed twice a day at 8:00 and 17:00, with a daily ration of approximately 4% of the body weight. The total biomass of each tank was determined by weighing all surviving crayfish at 7-day intervals throughout the 8-week feeding trial. Approximately 50% of the water was exchanged from each tank on a daily basis along with the siphoning of fecal matter from the bottom. The water parameters were consistent with the acclimation conditions.

### 2.5. Sample Collection

After the 8-week feeding trial, the crayfish were fasted for 24 h prior to the sample collection. All animals were inspected for external health status; only individuals with intact appendages, no visible lesions or discoloration, and active feeding and swimming behavior were selected for the trial. The crayfish in each tank were weighed and counted for the subsequent calculation of growth performance indices. All crayfish in each tank were sampled. Hemolymph was collected from the third pleopod using a 1 mL syringe prefilled with an equal volume of anticoagulant solution (26.3 g/L NaCl, 18.0 g/L glucose, 6.3 g/L citric acid monohydrate, 6.7 g/L disodium citrate dihydrate, and 2.9 g/L EDTA) [29]. The hemolymph was subsequently centrifuged at 4 °C (3000 rpm, 10 min) in order to obtain the anticoagulated hemolymph plasma. After anticoagulated hemolymph plasma collection, the crayfish were dissected on ice, and the hepatopancreas, muscle, and intestinal tissues were harvested. All the samples were snap-frozen in liquid nitrogen and stored at −80 °C until further use. During testing, three crayfish tissue samples were randomly selected from each tank and pooled for testing. The growth performance parameters were calculated as shown in Equations (1)–(3).

Survival rate (SR, %) = (Final crayfish number/Initial crayfish number) × 100(1)

Weight gain rate (WG, %) = (Final body weight − Initial body weight)/Initial body weight × 100(2)

Specific growth rate (SGR, %/day) = [ln(Final body weight) − ln(Initial body weight)] × 100/days(3)

### 2.6. Proximate Composition Analysis

The muscle tissues and the diets were subjected to proximate composition analysis in accordance with the AOAC methods [30]. Six crayfish were selected from each rearing tank, and their muscle tissue was collected and combined to analyze their muscle composition. The crude protein content was determined using the Kjeldahl method (AOAC 984.13, nitrogen × 6.25) (Kjeltec, Foss, Denmark); the crude lipid content was analyzed by the Soxhlet extraction method (AOAC 920.39, SZF-06A, Hongji, Shanghai, China); the moisture content was measured gravimetrically using an oven (AOAC 934.01, Shanghai Yiheng Scientific Instrument Co., Shanghai, China) at 105 °C; and the ash content was determined using a muffle furnace (AOAC 942.05, SX2–2.5–10, Yiheng, Shanghai, China) at 550 °C for 10 h).

### 2.7. Morphological Analysis

The hepatopancreas samples from each group were fixed in 4% paraformaldehyde (Biosharp Life Sciences, Hefei, China) for 48 h. All samples were dehydrated in graded ethanol (Xilong Science Co., Ltd., Shantou, China) concentration series and paraffin-embedded. Continuous sections of 5 μm thickness were prepared using a rotary microtome and were subsequently stained with hematoxylin and eosin (H&E) (G1005, Servicebio, Wuhan, China) [31]. The sections were examined under an upright microscope (Nikon Eclipse E100, Tokyo, Japan), and multifield digital images were acquired using an image analysis system (NIS-Elements D 4.6; 400×, Melville, NY, USA) for comprehensive documentation of the tissue architecture.

### 2.8. Biochemical Parameter Analysis

The activities of alkaline phosphatase (AKP, A059-2-2), acid phosphatase (ACP, A060-2-2), the content of malondialdehyde (MDA, A003-2-2), and the activities of glutathione peroxidase (GPx, A005-1-2), catalase (CAT, A007-2-1), superoxide dismutase (SOD, A001-3-2), along with the total antioxidant capacity (T-AOC, A015-2-1) in the hepatopancreas and hemolymph were determined using commercial assay kits, according to the instructions provided by the Nanjing Jiancheng Bioengineering Institute (http://www.njjcbio.com/ (accessed on 12 July 2024)). The phenol oxidase (PO, mL-E-23524) activity was determined using a commercial kit provided by Suzhou Grace Biotechnology Co., Suzhou, China, following the manufacturer-provided instructions. The digestive enzyme activities of trypsin (TPS, A080-2-2), amylase (AMS, C016-1-1), and lipase (LPS, A054-2-1) were also determined using commercial kits, following the manufacturer-provided instructions of the Nanjing Jiancheng Bioengineering Institute, Nanjing, China. The volume of the anticoagulant was taken into account and calculated.

### 2.9. Gene Expression

The total RNA from the hepatopancreas was extracted using the Total RNA Isolation Kit (Vazyme, Nanjing, China) and treated with RNase-free DNase I (Takara, Dalian, China). The RNA concentration and purity were determined using a NanoDrop One spectrophotometer (Thermo Fisher Scientific, Madison, WI, USA), and the integrity of the total RNA was verified by 1% agarose gel electrophoresis [29].

The complementary DNA (cDNA) was synthesized via reverse transcription using the PrimeScript RT Reagent Kit with gDNA Eraser (Takara, Dalian, China). The synthesized cDNA was stored at −20 °C until qRT-PCR analysis. qRT-PCR was performed on a LightCycler^®^ 96 Real-Time PCR System using the SYBR^®^ Green qPCR Master Mix. The primer pairs were designed using the Primer Premier v5 software based on the transcriptome sequences (Table 2). The PCR protocol followed that described in a previous study [29]. The relative gene expression levels were calculated using the 2^−ΔΔCt^ method.

### 2.10. Gut Microbiome Analysis

Three crayfish were selected from each rearing tank, and their guts were collected and combined for gut microbiome analysis. The total genomic DNA of the microbial communities was extracted using a commercial DNA extraction kit (Omega Biotech, Norcross, GA, USA), according to the manufacturer’s instructions. The DNA quality was evaluated by 1% agarose gel electrophoresis, and the DNA concentration and purity were determined using a NanoDrop 2000 spectrophotometer. The *16S rRNA* gene was amplified from the extracted genomic DNA using the universal primers 338F (5′-ACTCCTACGGGAGGCAGCA-3′) and 806R (5′-GGACTACHVGGGTWTCTAAT-3′). Purification, quantification, and sequencing were performed using an Illumina NextSeq 2000 platform. The detailed PCR program was described previously [32]. The libraries were constructed using the NEXTFLEX Rapid DNA-Seq Kit. The raw sequencing data were assembled using the FLASH software (v1.2.11). The effective tags were clustered using the UPARSE software (v7.0.1090). Sequences with ≥97% similarity were assigned to the same operational taxonomic unit (OTU). The alpha diversity indices (Chao1, Shannon, Simpson, and ACE) were calculated using the Mothur software (v1.30.2). The beta diversity analysis was conducted using the “vegan” package (version 4.2.1) in R, based on the Bray–Curtis distance. Since the treatment groups shared a recirculating water source, the findings regarding gut microbiota differences in this study may have been influenced by microbial exchange in the water; therefore, the microbiota data should be interpreted as indicative of associations rather than as independent evidence of causation.

### 2.11. Aeromonas Hydrophila Toxicity Test

The LC_50_ of *A. hydrophila* against crayfish over 72 h was determined to be 1 × 10^8^ CFU/crayfish during the preliminary trials. Activated *A. hydrophila* was inoculated into LB liquid medium for culture and incubated at 180 rpm for 24 h with agitation. The bacterial liquid was then centrifuged at 4 °C (4000 rpm, 10 min). The resulting bacterial suspension was diluted to 1 × 10^8^ CFU/mL with PBS (0.1 mM, pH 7.4) for use. Following 56 days of feeding, 30 healthy individuals were randomly selected from each group and were intramuscularly injected with 15 μL of the bacterial suspension. The injected crayfish were subsequently reared in three tanks. The mortality was recorded over a period of 96 h, and the cumulative mortality rate was calculated.

### 2.12. Statistical Analysis

Three pools of samples from each tank were tested for these indicators. All the experimental data are expressed as the mean ± standard deviation (mean ± SD). The differences among the groups were analyzed by one-way analysis of variance (ANOVA), using the SPSS 20.0 statistical software, followed by Tukey’s multiple comparison test. *p* < 0.05 indicated statistical significance.

## 3. Results

### 3.1. AO Analysis

LC-MS analysis of AO employed in the study indicated the presence of various chemicals. The compounds compared and identified by a previous method [33] and their characteristic information are presented in Table S1. Based on the calibration curve (Figure S1), the total flavonoid and total phenolic contents of the AO were 2.36 ± 0.17 mg/g and 13.98 ± 1.19 mg/g, respectively.

### 3.2. Growth Performance

The effects of AO supplementation on the growth performance of *C. quadricarinatus* are presented in Table 3. Compared to the control group, both the SGR and WG were significantly higher in the AO3 and AO4 groups (*p* < 0.05). Crayfish responded exponentially to graded dietary AO levels (Figure S2). The best WG and SGR were observed at 3.65 and 3.93 g/kg dietary AO levels, respectively. No significant differences were observed in the SR among the groups (*p* ˃ 0.05).

### 3.3. Muscle Composition

The effects of AO supplementation on the muscle composition of *C. quadricarinatus* are presented in Table 4. As compared to the control group, the crude lipid content was significantly reduced in the AO3 and AO4 groups (*p* < 0.05), whereas the crude protein content was significantly elevated in the AO2, AO3, and AO4 groups (*p* < 0.05). No significant differences in the moisture or ash contents were observed among the groups (*p* ˃ 0.05).

### 3.4. Morphological Structure of Hepatopancreas

**Figure 1:**
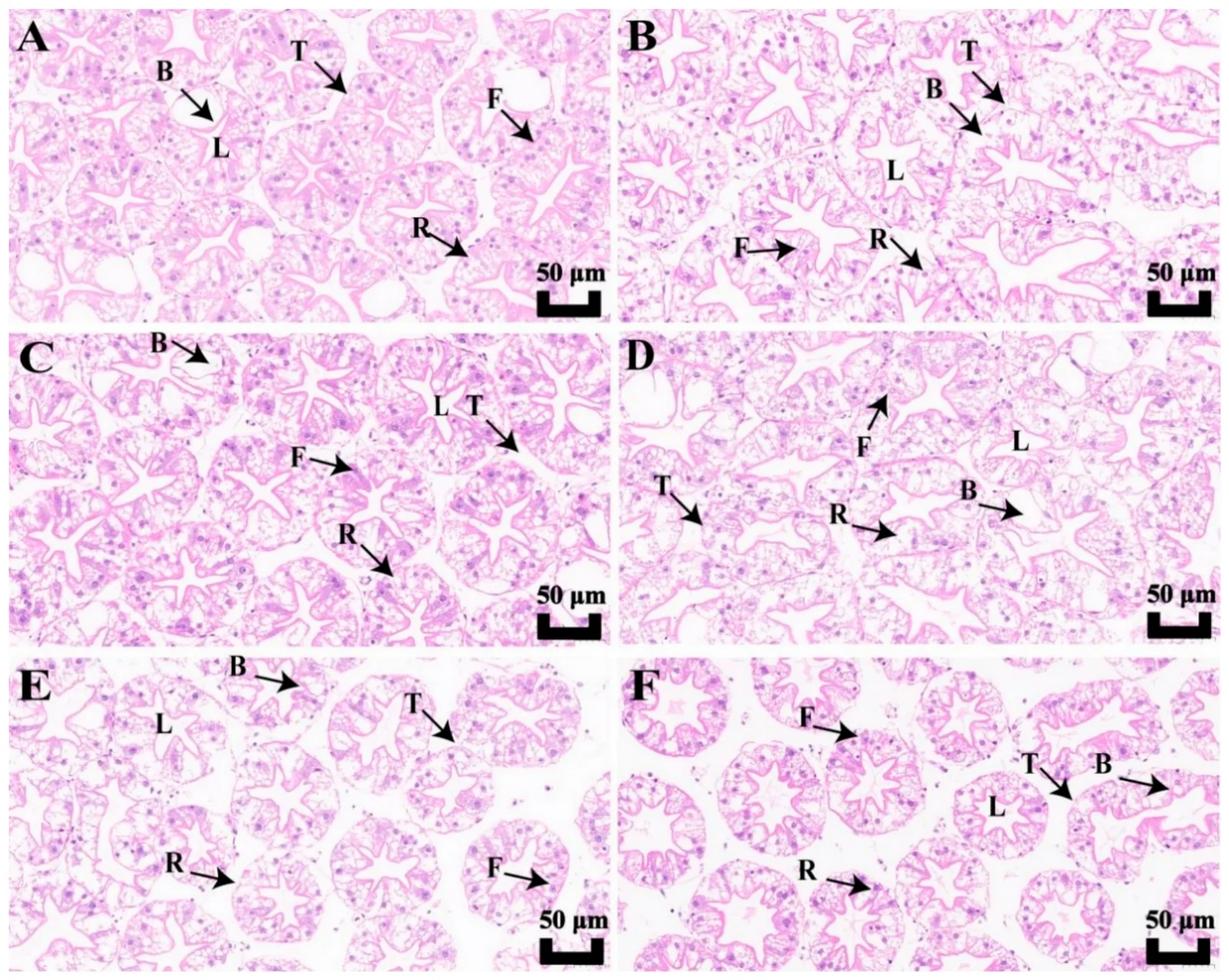
Histological changes in the hepatopancreas of *Cherax quadricarinatus* fed diets supplemented with different AO concentrations (original magnification 400×). (**A**) CG (0 g/kg); (**B**) AO1 (0.5 g/kg); (**C**) AO2 (1 g/kg); (**D**) AO3 (3 g/kg); (**E**) AO4 (5 g/kg); (**F**) AO5 (7 g/kg). T: Tubule, L: Lumen, R: R-cells, F: F-cells, B: B-cells.

The morphological features of the hepatopancreas are shown in Figure 1. The control group exhibited a compact arrangement of hepatic tubules, with clearly defined lumina and no evidence of tubular expansion or atrophy. As compared to the control group, the AO-treated groups demonstrated no significant morphological alterations in the hepatopancreas.

### 3.5. Antioxidant and Immune Parameters

**Figure 2:**
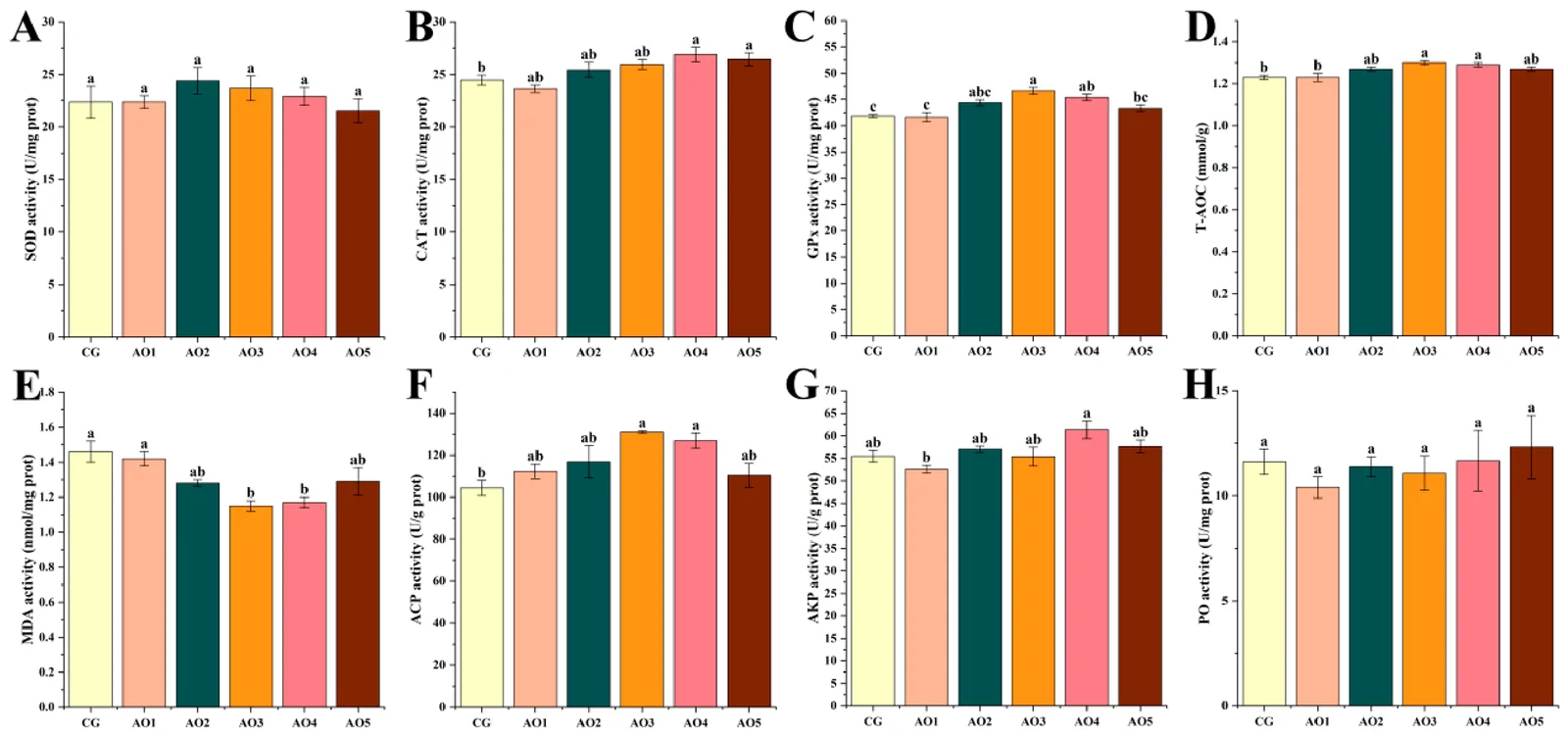
Effects of AO supplementation in diet on antioxidant and immune parameters of hepatopancreas in *Cherax quadricarinatus*. (**A**) SOD activity, (**B**) CAT activity, (**C**) GPx activity, (**D**) T-AOC, (**E**) MDA content, (**F**) ACP activity, (**G**) AKP activity, (**H**) PO activity. Data are presented as means ± SD (n = 3 tanks per treatment, with 40 crayfish per tank initially). Different letters indicate significant differences (*p* < 0.05).

The effects of AO supplementation on the antioxidant and immune parameters in the hepatopancreas of the crayfish are presented in Figure 2. Compared to the control group, the CAT activity was significantly elevated in the AO4 and AO5 groups (*p* < 0.05; Figure 2B). Similarly, the GPx activity (Figure 2C), ACP activity (Figure 2F), and T-AOC (Figure 2D) in the hepatopancreas (*p* < 0.05) significantly increased in the AO3 and AO4 groups, whereas the MDA content (Figure 2E) significantly reduced (*p* < 0.05). No significant differences in the activities of SOD (Figure 2A), PO (Figure 2H), or AKP (Figure 2G) were observed among the AO-treated groups as compared to the control group (*p* ˃ 0.05).

**Figure 3:**
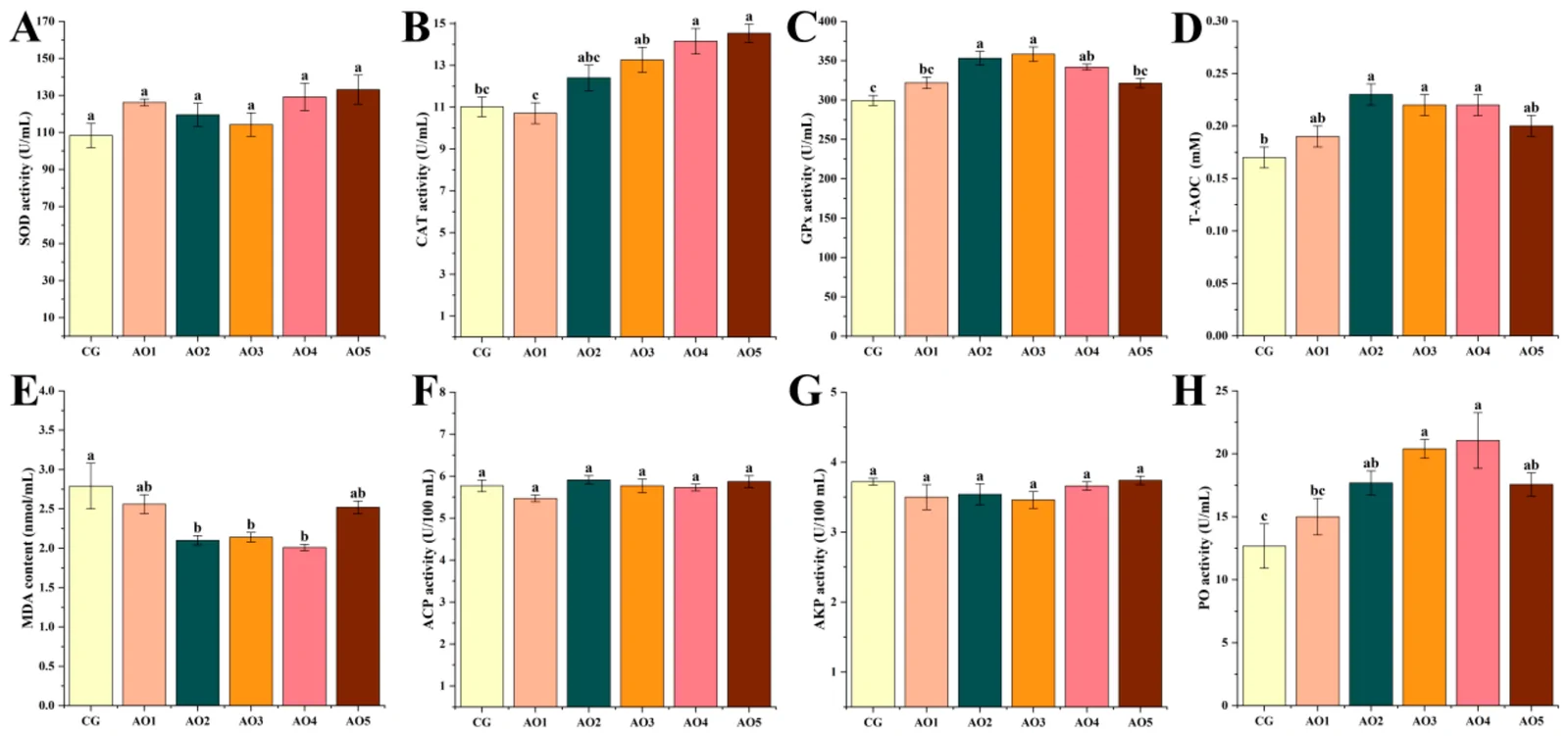
Effects of AO supplementation in diet on hemolymph antioxidant and immune parameters in *Cherax quadricarinatus*. (**A**) SOD activity, (**B**) CAT activity, (**C**) GPx activity, (**D**) T-AOC, (**E**) MDA content, (**F**) ACP activity, (**G**) AKP activity, and (**H**) PO activity. Data are presented as means ± SD (n = 3 tanks per treatment, with 40 crayfish per tank initially). Different letters indicate significant differences (*p* < 0.05).

The effects of AO supplementation on the hemolymph antioxidant and immune parameters in the crayfish are presented in Figure 3. Compared to the control group, the GPx activity and the T-AOC in the AO2, AO3, and AO4 groups were significantly elevated (*p* < 0.05, Figure 3C,D), whereas the MDA content was significantly reduced (*p* < 0.05, Figure 3E). The CAT activity significantly increased in the AO3, AO4, and AO5 groups as compared to the control group (*p* < 0.05, Figure B). PO activity was significantly elevated in the AO2, AO3, AO4, and AO5 groups as compared to the control group (*p* < 0.05, Figure 3H). No significant differences in the activities of SOD, ACP, or AKP were observed among the groups (*p* ˃ 0.05, Figure 3A,F,G).

### 3.6. Digestive Ability

The effects of AO supplementation on the digestive enzyme activities in the intestine of the crayfish are presented in Table 5. Trypsin and lipase activities were significantly higher in the AO3 and AO4 groups than in the control group (*p* < 0.05). No significant differences in the amylase activity were observed among the groups (*p* ˃ 0.05).

### 3.7. Gene Expression

**Figure 4:**
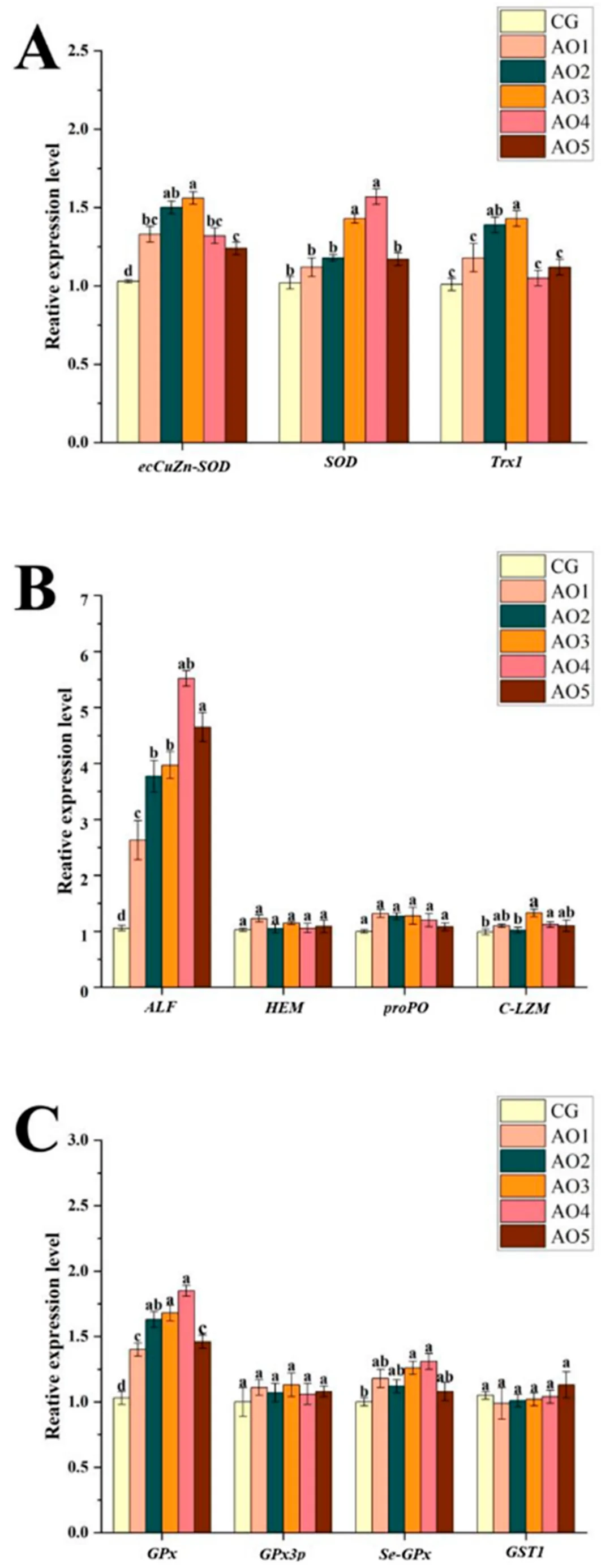
Effects of different AO concentrations on antioxidant and immune-related gene expression levels in *Cherax quadricarinatus*. (**A**): Antioxidant-related genes; (**B**): immune-related genes; (**C**): glutathione metabolism-related genes. *ecCu, Zn-SOD*: extracellular copper/zinc superoxide dismutase; *SOD*: superoxide dismutase; *Trx1*: thioredoxin 1; *ALF*: anti-liposaccharide factor; *HEM*: hemocyanin; *proPO*: prophenoloxidase; *C-LZM*: C-type lysozyme; *GPx*: glutathione peroxidase; *GPx3P*: glutathione peroxidase 3 precursor; *Se-GPx*: selenium-dependent glutathione peroxidase; *GST1*: glutathione S transferase D1. Data are presented as means ± SD (n = 3 tanks per treatment, with 40 crayfish per tank initially). Different letters indicate significant differences (*p* < 0.05).

The effects of AO supplementation on the mRNA expression levels of the antioxidant-related genes in the hepatopancreas are shown in Figure 4A. As compared to the control group, the expression of *ecCu, Zn-SOD* exhibited an initial increase followed by a decrease with the increasing dietary AO concentration. The *SOD* expression was significantly elevated in the AO3 and AO4 groups (*p* < 0.05), whereas the *Trx1* expression significantly increased in the AO2 and AO3 groups (*p* < 0.05).

The expression levels of the immune-related genes in the hepatopancreas are shown in Figure 4B. As compared to the control group, dietary AO supplementation significantly upregulated the expression of *ALF* (*p* < 0.05), with the AO4 group demonstrating the highest expression level. The relative mRNA expression of *C-LZM* was significantly higher in the AO3 and AO4 groups compared to the control group (*p* < 0.05). However, dietary AO had no significant effect on the expression of *proPO* or *HEM* (*p* ˃ 0.05).

Figure 4C illustrates the effect of dietary AO supplementation on the expression levels of the glutathione metabolism-related genes in the hepatopancreas. As compared to the control group, the expression of *GPx* exhibited an initial increase followed by a decrease with the increasing dietary AO concentration. The *Se-GPx* expression was significantly elevated in the AO3 and AO4 groups compared to the control group (*p* < 0.05). No significant differences were observed in the expression of *GPx3P* or *GST1* among the groups (*p* ˃ 0.05).

### 3.8. Gut Microbiota

**Figure 5:**
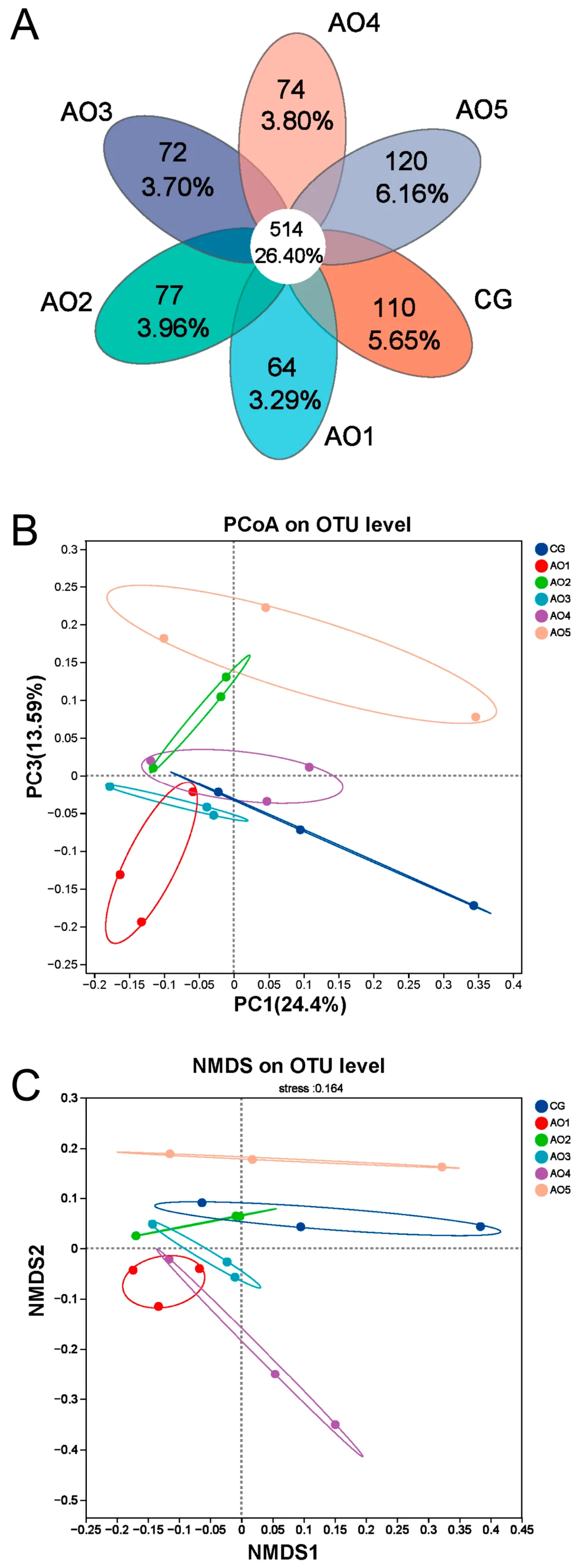
Differences in gut microbiota diversity among *Cherax quadricarinatus* at different AO concentrations. (**A**) Venn diagram analysis of shared OTUs and unique OTUs among six groups; (**B**) Principal coordinates analysis (PCoA); (**C**) Non-metric Multidimensional Scaling (NMDS). Note: n = 3 tanks per treatment, with 40 crayfish per tank initially.

Based on the Venn diagram analysis (Figure 5A), a total of 1026 OTUs were identified across the six groups, of which 514 OTUs were common to all the six experimental groups. Each group (CG, AO1, AO2, AO3, AO4, and AO5) contained 110, 64, 77, 72, 74, and 120 unique OTUs, respectively.

Principal coordinates analysis (PCA) was conducted in order to evaluate the similarity matrix among the samples (Figure 5B). PC1 accounted for 24.4% of the total variance, whereas PC3 accounted for 13.59%. Additionally, the NMDS analysis further revealed the degree of variation among the samples (Figure 5C). The alpha diversity results were presented in Table S2. There were no significant differences in Shannon, ACE, Chao1 and Simpson parameters among the experimental groups.

**Figure 6:**
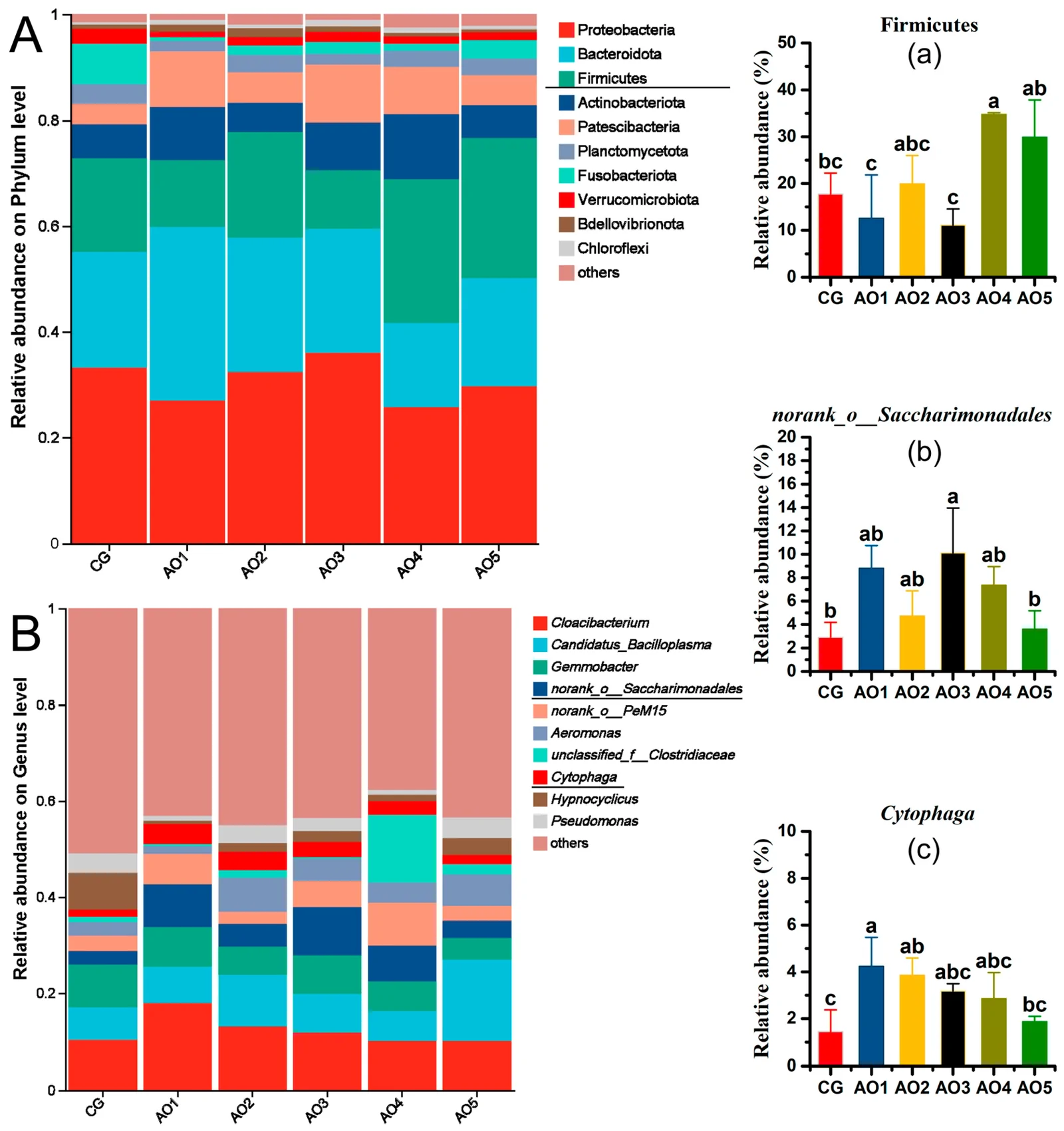
Differences in gut microbiota composition among all groups in *Cherax quadricarinatus*. (**A**) Relative abundance of gut microbiota at the phylum level; (**B**) relative abundance of gut microbiota at the genus level. The species on the slash are shown on the right side of the figure. (**a**) Firmicutes; (**b**) *norank_o_Saccharimonadales*; (**c**) *Cytophaga*. Different letters indicate significant differences (*p* < 0.05).

Figure 6A shows the top 10 phyla in the intestinal microbiota of *C. quadricarinatus*. Proteobacteria was the most dominant phylum, followed by Bacteroidetes and Firmicutes. As compared to the control group, Firmicutes abundance significantly increased in the AO4 group (*p* < 0.05). At the genus level, *Cloacibacterium* was the most dominant genus, followed by *Candidatus_Bacilloplasma* and *Gemmobacter* (Figure 6B). As compared to the control group, the abundance of the genus *Cytophaga* significantly increased in the AO1 and AO2 groups, whereas *norank_o_Saccharimonadales* was significantly elevated in the AO3 group (*p* < 0.05).

### 3.9. Disease Resistance

**Figure 7:**
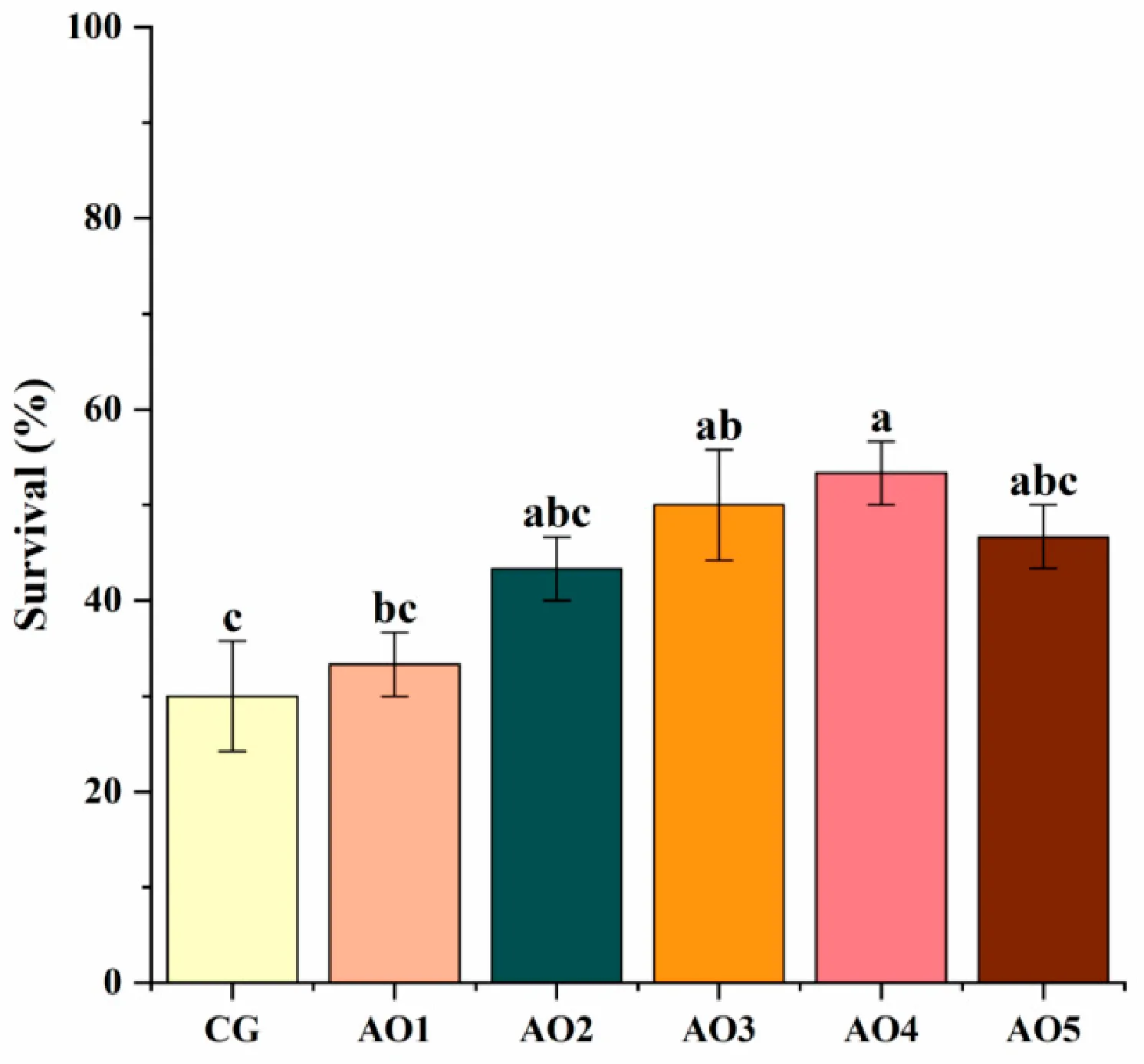
Effects of different AO concentrations on survival rate of *Cherax quadricarinatus* infected with *A. hydrophila*. Data are presented as means ± SD (n = 3 tanks per treatment, with 40 crayfish per tank initially). Different letters indicate significant differences (*p* < 0.05).

The survival rate of the crayfish after 96 h of challenge with *A. hydrophila* is shown in Figure 7. As compared to the control group, dietary supplementation with 3–5 g/kg AO significantly improved the survival rate of crayfish (*p* < 0.05).

## 4. Discussion

### 4.1. Nutritional and Functional Quality of the Experimental Feeds

Numerous studies have demonstrated that extracts derived from medicinal plants can directly or indirectly enhance the growth performance [34], antioxidant capacity [35], immune function [36], and disease resistance [37] of aquatic animals. Our previous investigation revealed that dietary supplementation with sharp-leaf galangal (*Alpinia oxyphylla*) fruit extract significantly improved growth performance, gut microbiota composition, immune responses, and disease resistance in red claw crayfish [26]. Given that *A. oxyphylla* and *A. officinarum* belong to the same genus and share a spectrum of structurally related polyphenolic compounds with comparable bioactivities, it is plausible that galangal extract may exert analogous beneficial effects. Galangal extract has been shown to exhibit antibacterial, antioxidant, and anti-inflammatory properties [38]. Furthermore, galangal extract enhances the antioxidant capacity, immunity, and disease resistance of aquatic animals [23,24]. The present study, therefore, evaluates the effects of dietary galangal extract on the growth performance, immune response, digestive capacity, gut microbiota composition, and disease resistance of red claw crayfish.

### 4.2. Growth and Body Performance

Plant extracts, such as berberine, have been reported to significantly improve the growth performance of *Pelteobagrus fulvidraco* [39]. Grape seed proanthocyanidin extracts have been shown to enhance the growth performance of the common carp (*Cyprinus carpio*) [40], whereas oregano essential oil has been reported to promote the growth of both the common carp and the Nile tilapia (*Oreochromis niloticus*) by stimulating digestive enzyme activity and modulating gut microbiota [41,42]. In the present study, dietary supplementation with 3–5 g/kg of AO significantly increased both the WG and the SGR of the red claw crayfish. Recent research has demonstrated that dietary supplementation with a plant extract blend significantly increases the growth performance and elevates the activities of the digestive enzymes, including protease, amylase, and lipase [43]. This observation is consistent with the findings of the present study. The dietary supplementation AO not only enhanced the growth performance but also increased selected digestive enzyme activities of the crayfish. A previous study has suggested that the enhanced growth performance is closely associated with the elevated digestive enzyme activity [44]. This observation was further supported in the present study. Survival rates of red claw crayfish in this study were observed to range from 85.83% to 91.67%. This relatively low survival is most plausibly explained by cannibalistic behavior, wherein vulnerability is markedly elevated during the immediate post-molt period, when soft-shelled animals are easily preyed upon by their own kind.

In the present study, dietary supplementation with AO did not significantly affect the morphology of the hepatopancreas in crayfish. Previous studies have reported that dietary supplementation with plant extracts, such as *Radix bupleuri* root extract [29] and *Silybum marianum* extract [45], does not significantly alter the hepatopancreatic tissue of aquatic animals. These findings indicate that dietary AO did not induce hepatopancreatic damage. Dietary AO supplementation significantly elevated crude protein content while reducing the crude lipid content in crayfish muscle, thereby suggesting that AO may modulate the lipid metabolism and protein deposition in crayfish [46,47]. Previous studies have reported that feeding plant extracts improves the muscle composition of aquatic animals [48], which is consistent with the findings of the present study. However, the information concerning the effects of dietary plant extracts on the nutritional metabolism of the crustacean muscle has remained limited, and the underlying mechanisms are incompletely understood, warranting further investigation.

### 4.3. Antioxidant and Immune Responses

Excessive production of reactive oxygen species (ROS) leads to tissue peroxidation [49], and organisms eliminate the excess ROS through their antioxidant defense systems [50]. The antioxidant defense system, which employs multiple enzymes to eliminate the excess ROS, is essential for the maintenance of normal physiological functions. In biological systems, MDA, the end product of polyunsaturated fatty acid peroxidation, serves as a direct indicator of oxidative damage to the cellular and organelle membranes [51,52]. SOD, CAT, and GPx are key enzymes involved in ROS scavenging and constitute the core components of the antioxidant defense system. The variations in their activities are widely used to assess the antioxidant defense capacity of an organism [53,54]. T-AOC reflects an organism’s resistance to external stressors and the metabolic state of intracellular free radicals, thereby serving as a comprehensive indicator for assessment of the antioxidant defense function and the overall organismal health [55]. In the present study, dietary AO supplementation significantly increased the GPx and CAT activities along with the T-AOC of crayfish while reducing the MDA content. These findings indicate that AO effectively activates the endogenous antioxidant enzyme system, thereby enhancing the crayfish’s capacity to scavenge ROS. Consequently, the lipid peroxidation damage to the cellular and organelle membranes is attenuated, and the normal metabolic and secretory functions of the crayfish are preserved. These findings are consistent with previous reports in the Pacific white shrimp (*Litopenaeus vannamei*) and abalone (*Haliotis discus hannai*) [56,57]. In the present study, the changes in the antioxidant gene expression were consistent with the alterations in enzyme activity, suggesting that the variations in enzymatic activity may be transcriptionally regulated. These findings corroborate the conclusions of several recent investigations, which collectively highlight the efficacy of plant extracts in modulating the immune systems of aquatic animals through multi-target regulation. For instance, *Lycium barbarum* polysaccharides activate the Nrf2–Keap1 signaling pathway, thereby upregulating the downstream phase II detoxification enzymes and antioxidant enzymes, including GPx and CAT; this provides a potential molecular mechanism that may underlie the antioxidant-enhancing activity of the plant extract [58]. Another study on the black tiger shrimp has reported that polyphenol-rich plant extracts significantly reduce tissue lipid peroxidation (LPO) while increasing the activities of SOD, CAT, GPx, and glutathione S-transferase (GST), thereby enhancing the antioxidant status of the silver catfish (*Rhamdia quelen*) [59]. Previous research has indicated that antioxidant gene expression may be closely associated with the regulation of metabolic pathways [60,61]. This observation suggests that dietary AO may modulate the metabolism and the associated signaling pathways in crayfish, thereby influencing its antioxidant capacity. However, the precise underlying mechanisms warrant further investigation.

ACP and AKP contribute to the elimination and neutralization of microbial pathogens. They play a critical role in nonspecific immune responses and serve as important indicators of the disease resistance of the host [62,63]. Active PO enhances pathogen recognition, encapsulation, and melanization, thereby comprehensively strengthening the nonspecific immune defense system of the host [64]. In the present study, supplementing feed with 3–5 g/kg of AO significantly enhanced the activities of ACP in the hepatopancreas and PO in the hemolymph of crayfish. Accumulating evidence in red claw crayfish has confirmed that dietary supplementation with various phytogenic bioactive compounds, including sharp-leaf galangal (*Alpinia oxyphylla*) fruit extract [26], *Eucommia ulmoides* leaf extract [65], glycerol monolaurate [66], and coumarin [67], consistently elevates the activities of ACP and PO, underscoring the immunostimulatory potential of plant-derived additives in this species. Previous studies have demonstrated that dietary plant extracts can enhance ACP and PO activities in aquatic animals [68,69,70]. Moreover, AO has been shown to activate the hemolymph peroxidase activity in a dose-dependent manner, thereby enhancing resistance to vibrio infection [71]. The significant enhancement in enzyme activities indicates that AO may reinforce innate immune defense capabilities at the cellular level by elevating phosphatase and phenoloxidase activities, thereby facilitating the digestion and clearance of the phagocytosed pathogens [72].

The immune factors in crustaceans play multiple roles in nonspecific immune regulation and are essential for the maintenance of normal physiological metabolism in the host [73]. ALF and LZM are key antimicrobial peptides (AMPs) in crustaceans and play a crucial role in innate immunity [74]. ALF is an alkaline protein with antibacterial activity that binds to and neutralizes lipopolysaccharides (LPSs) [75]. The proPO system is closely associated with PO activity, and the activation of proPO protects the host from pathogenic bacteria [76]. Beyond its primary role in pathogen melanization and encapsulation, the activation of the proPO system also contributes to broader immune regulatory networks by producing immunomodulatory intermediates, such as quinones [77]. In the present study, dietary AO supplementation induced the expression of *ALF* and *C-LZM*. This finding is corroborated by a growing body of evidence in red claw crayfish, demonstrating that various phytogenic compounds, including dietary quercetin (a plant flavonoid) [78], coumarin (a natural plant-derived compound) [67], and *Alpinia oxyphylla* fruit extract [26], consistently upregulate the mRNA expression of immune-related genes such as *ALF*, *proPO*, *JAK*, and *STAT*. The induced expression of AMPs has been reported to enhance disease resistance in aquatic animals [79]. Previous studies have shown that dietary plant extracts can enhance disease resistance by inducing the expression of immune factors [80,81]. However, research on the immunomodulatory mechanisms of AO in aquatic animals remains limited, and further investigation is warranted in order to elucidate the specific mechanisms in the future.

### 4.4. Gut Microbiota

The composition of the gut microbiota is crucial for the growth and development of the host, and it is primarily shaped by the living environment and feeding habits [82]. The present study demonstrated that the gut microbiota of the red claw crayfish was predominantly composed of the phyla Proteobacteria, Firmicutes, and Bacteroidota, which is consistent with the previous reports [83]. Firmicutes play a key role in maintaining the integrity of the intestinal barrier and host health [84]. In the present study, the abundance of Firmicutes was elevated in the AO4 group. Many *Cytophaga*-like bacteria are encapsulated within a thin mucous layer, which facilitates cell adhesion to the substrate surfaces while permitting translational movement. These bacteria commonly exhibit lytic activity, which appears to be a shared characteristic of the bacteria within the genus *Cytophaga* [85]. *norank_o_Saccharimonadales* has been implicated in substance metabolism [86]. Dietary AO supplementation altered the composition of these microorganisms, thereby potentially influencing the digestive capacity of crayfish. These findings indicate that dietary AO modulates the gut microbial composition of crayfish. Previous studies have shown that dietary plant extracts significantly alter the gut microbiota structure of crustaceans [87,88], which is consistent with the findings of the present study.

### 4.5. Disease Resistance

Owing to its motility and robust environmental adaptability, *A. hydrophila* secretes virulence factors, such as hemolysin, proteases, and enterotoxins, which enable it to cause septicemia, skin ulceration, and intestinal lesions in several aquatic animals, and has become a leading pathogen responsible for bacterial disease outbreaks in aquaculture [89]. A previous study has indicated that AO can alter the hydrophobicity, fluidity, and permeability of the bacterial cell membrane, thereby exerting an antibacterial effect. In the present study, dietary supplementation with 3–5 g/kg of AO significantly improved the survival rate of the crayfish challenged with *A. hydrophila*. Furthermore, a previous investigation reported that AO activated the expression of the host immune system, thereby enhancing disease resistance [90], which is consistent with the findings of the present study. The enhanced disease resistance is closely linked to the antioxidant capacity and the immune function of the host [91,92]. In the present study, dietary AO supplementation significantly enhanced the antioxidant capacity and the expression of immune factors in the red claw crayfish, which may account for the improved survival rate of the crayfish following challenge with *A. hydrophila*.

## 5. Conclusions

In conclusion, dietary supplementation with 3–5 g/kg of AO significantly enhanced the growth performance, immunity, and disease resistance of red claw crayfish. Dietary AO also was associated with altered composition or enzyme activity in the intestine. Notably, AO supplementation markedly elevated the activities of antioxidant enzymes and immune-related enzymes in the hepatopancreas and hemolymph, reflecting an augmented nonspecific immune defense capacity. Furthermore, AO supplementation induced the expression of the antioxidant- and immune-related genes in the hepatopancreas, without causing any structural damage to the hepatopancreatic tissue. Collectively, these findings highlight that AO may warrant evaluation as a candidate phytogenic additive. Future studies should focus on the refinement of its cross-species application protocols and on the evaluation of its long-term effects on the health status and disease tolerance under commercial farming conditions.

## Figures and Tables

**Table 1:** Proximate composition of the basal diet for *Cherax quadricarinatus* (%).

Ingredients	CG	AO1	AO2	AO3	AO4	AO5
	0	0.5	1	3	5	7
Fish meal	26	26	26	26	26	26
Soybean meal	15	15	15	15	15	15
Blood meal	2	2	2	2	2	2
Peanut meal	18	18	18	18	18	18
Wheat flour	12	12	12	12	12	12
Corn starch	10	10	10	10	10	10
Fish oil	5	5	5	5	5	5
Cellulose	4.5	4.45	4.4	4.2	4	3.8
Shell powder	1	1	1	1	1	1
Ca(H_2_PO_4_)_2_	1.5	1.5	1.5	1.5	1.5	1.5
Sodium carboxymethyl cellulose	1	1	1	1	1	1
Sodium alginate	2	2	2	2	2	2
Vitamin premix ^1^	1	1	1	1	1	1
Mineral premix ^2^	1	1	1	1	1	1
*Alpinia officinarum* extract	0	0.05	0.1	0.3	0.5	0.7
Nutrient levels						
Crude protein	28.09	28.88	29.53	28.21	28.35	29.46
Crude lipid	10.35	10.84	10.12	9.96	10.18	10.46
Moisture	11.15	10.64	11.19	11.84	11.53	11.04

^1^ Vitamin premix (g/kg diet): biotin (45 g), folic acid (0.3 g), nicotinic acid (18 g), calcium pantothenate (7 g), vitamin C (8 g), vitamin B_12_ (0.02 g), vitamin B_6_ (5 g), vitamin B_2_ (5 g), vitamin B_1_ (2 g), vitamin K_3_ (2 g), vitamin E (18 IU), vitamin D_3_ (1000 IU), and vitamin A (9000 IU). ^2^ Mineral premix (g/kg diet): Na_2_SeO_3_ (0.02 g), CoSO_4_·7H_2_O (0.1 g), KI (0.04 g), FeSO_4_·H_2_O (25 g), MnSO_4_·H_2_O (15 g), ZnSO_4_·H_2_O (25 g), and CuSO_4_·5H_2_O (2 g).

**Table 2:** Primers used for qRT-PCR.

Genes	Primers (5′−3′)	Efficiency Values	Product Size
*Se-GPx*	F: CGTCAGAGGGAAAGGGAAGT	1.01	163
	R: GAGAATGCCACTGAGTCGGA		
*GPx3P*	F: CTAATCGGTAGGAATGGCAAGC	1.02	145
	R: CGCCTGGGTCTATGGGAAC		
*GST1*	F: ACCGTAGGATTGCGTTGCT	1.08	113
	R: GGAAAAGTCTGATTGGTTGCC		
*GPx*	F: CAAGGTGGTGTTGGTGGTCA	1.03	228
	R: TCAAAGAAGGTCATCAGAGGCT		
*C-LZM*	F: GCAACAGGAACGGTAGCAAG	1.07	190
	R: GCCACCCAAGCGGAATAT		
*proPO*	F: GCTTCACTAAGTCCCAACGCT	1.03	119
	R: TCCCATCACTCCAATCTCCTC		
*HEM*	F: ACATAGCAAGGACTGGCAAAC	1.03	149
	R: TGACCTCGTAAAGTGGTGGAA		
*Trx1*	F: CATTAGCGCCAGAGAAGGAC	1.04	142
	R: ATGTAGCCATGGAACACCAG		
*ALF*	F: ACAGAGCAACGCACAGATTACA	1.05	176
	R: TCCAGCCAGGGCAATACAT		
*SOD*	F: CAAAATTCCCTTGCCAGCAC	1	124
	R: CCTGGTTCCAGCTTGATGTT		
*ecCu, ZnSOD*	F: TGTGGCGGCTCTGTGCT	0.96	120
	R: CCGACGATGGTGATTTCTCC		
*β-actin*	F: ATCACTGCTCTGGCTCCTGCTACC	1.08	148
	R: CGGACTCGTCGTACTCCTCCTTGG		

**Table 3:** Effects of different AO concentrations on growth performance of *Cherax quadricarinatus.*

	CG	AO1	AO2	AO3	AO4	AO5
IW (g)	0.86 ± 0.04	0.85 ± 0.06	0.82 ± 0.07	0.81 ± 0.05	0.80 ± 0.04	0.84 ± 0.05
FW (g)	3.26 ± 0.10 ^a^	3.41 ± 0.08 ^ab^	3.48 ± 0.05 ^ab^	3.57 ± 0.07 ^a^	3.51 ± 0.04 ^a^	3.37 ± 0.03 ^ab^
WG (%)	266.49 ± 11.59 ^b^	284.45 ± 7.54 ^ab^	290.48 ± 4.58 ^ab^	303.17 ± 6.93 ^a^	298.52 ± 3.52 ^a^	280.19 ± 3.48 ^ab^
SGR (%/day)	2.32 ± 0.06 ^b^	2.40 ± 0.04 ^ab^	2.43 ± 0.02 ^ab^	2.49 ± 0.03 ^a^	2.48 ± 0.02 ^a^	2.38 ± 0.02 ^ab^
SR (%)	86.67 ± 6.01	87.50 ± 1.44	85.00 ± 6.29	88.33 ± 5.83	85.83 ± 4.17	91.67 ± 1.67

Note: Data are presented as means ± SD (n = 3 tanks per treatment, with 40 crayfish per tank initially). Means in the same row with different superscripts are significantly different (*p* < 0.05).

**Table 4:** Effects of different AO concentrations on muscle composition of *Cherax quadricarinatus* (dry-mass basis).

	CG	AO1	AO2	AO3	AO4	AO5
Moisture (%)	77.33 ± 0.60	78.38 ± 0.24	77.25 ± 0.38	77.96 ± 0.55	77.96 ± 0.41	77.45 ± 0.12
Crude protein (%)	79.89 ± 0.54 ^b^	80.16 ± 0.51 ^b^	82.68 ± 0.49 ^a^	83.07 ± 0.28 ^a^	83.10 ± 0.45 ^a^	81.12 ± 0.43 ^ab^
Crude lipid (%)	2.35 ± 0.04 ^a^	2.19 ± 0.07 ^ab^	2.31 ± 0.09 ^a^	1.98 ± 0.05 ^b^	2.03 ± 0.05 ^b^	2.24 ± 0.06 ^ab^
Ash (%)	1.34 ± 0.08	1.38 ± 0.04	1.55 ± 0.07	1.38 ± 0.02	1.39 ± 0.05	1.89 ± 0.26

Note: The conversion factor for the Kjeldahl method is 6.25. Data are presented as means ± SD (n = 3 tanks per treatment, with 40 crayfish per tank initially). Means in the same row with different superscripts are significantly different (*p* < 0.05).

**Table 5:** Effects of different AO concentrations on digestive enzyme activity of *Cherax quadricarinatus.*

	CG	AO1	AO2	AO3	AO4	AO5
Amylase (U/mg prot)	33.18 ± 1.51	31.17 ± 1.26	34.53 ± 2.47	33.95 ± 2.45	33.01 ± 1.69	30.78 ± 2.42
Lipase (U/mg prot)	11.84 ± 0.94 ^b^	12.41 ± 0.53 ^b^	15.96 ± 0.80 ^ab^	17.14 ± 1.47 ^a^	18.40 ± 0.93 ^a^	13.02 ± 0.88 ^b^
Trypsin (U/mg prot)	27.28± 0.58 ^c^	29.5± 0.31 ^bc^	29.9± 0.92 ^bc^	33.67± 0.74 ^a^	31.91 ± 0.37 ^ab^	29.40± 0.69 ^bc^

Note: Data are presented as means ± SD (n = 3 tanks per treatment, with 40 crayfish per tank initially). Means in the same row with different superscripts are significantly different (*p* < 0.05).

## Data Availability

The data supporting the findings of this study are included within the article. Further inquiries can be directed to the corresponding author.

## Supplementary Materials

The following supporting information can be downloaded at: https://www.mdpi.com/article/10.3390/ani16162525/s1. Figure S1: Calibration curves for total flavonoids and total phenolics. Figure S2: Broken-line analysis of the relationship between the dietary AO and growth parameters of crayfish. Table S1: Characteristic information of compounds contained in AO. Table S2: Differences in gut microbiota alpha diversity among C. quadricarinatus at different AO concentrations.
